# New Medical Journal in Cincinnati, Ohio

**Published:** 1866-02

**Authors:** 


New Medical Journal in Cincinnati, Ohio.—We have the pleasure of adding to our exchange list *The Cincinnati Journal of Medicine*, January number, Vol. I, No. 1, being upon our table. It is published monthly, contains forty-eight pages, and has the following able editorial staff: George C. Blackman, M. D., Theophilus Parvin, M. D., Roberts Bartholow, M. D., Professors in the Medical College of Ohio.

